# “It is you, me on the team together, and my child”: Attending, resident, and patient family perspectives on patient ownership

**DOI:** 10.1007/s40037-020-00635-8

**Published:** 2020-12-02

**Authors:** Michelle E. Kiger, Holly S. Meyer, Lara Varpio

**Affiliations:** 1 Department of Pediatrics, Uniformed Services University of the Health Sciences, Bethesda, MD USA; 2 Department of Pediatrics, Wright-Patterson Medical Center, Dayton, OH USA; 3 Department of Medicine, Uniformed Services University of the Health Sciences, Bethesda, MD USA

**Keywords:** Patient ownership, Resident continuity clinics, Professionalism, Team-based care, Continuity of care

## Abstract

**Introduction:**

Patient ownership is an important element of physicians’ professional responsibility, but important gaps remain in our understanding of this concept. We sought to develop a theory of patient ownership by studying it in continuity clinics from the perspective of residents, attending physicians, and patients.

**Methods:**

Using constructivist grounded theory, we conducted 27 semi-structured interviews of attending physicians, residents, and patient families within two pediatric continuity clinics to examine definitions, expectations, and experiences of patient ownership from March–August 2019. We constructed themes using constant comparative analysis and developed a theory describing patient ownership that takes into account a diversity of perspectives.

**Results:**

Patient ownership was described as a bi-directional, relational commitment between patient/family and physician that includes affective and behavioral components. The experience of patient ownership was promoted by continuity of care and constrained by logistical and other systems-based factors. The physician was seen as part of a medical care team that included clinic staff and patient families. Physicians adjusted expectations surrounding patient ownership for residents based on scheduling limitations.

**Discussion:**

Our theory of patient ownership portrays the patient/family as an active participant in the patient–physician relationship, rather than a passive recipient of care. While specific expectations and tasks will vary based on the practice setting, our findings reframe the way in which patient ownership can be viewed and studied in the future by attending to a diversity of perspectives.

**Electronic supplementary material:**

The online version of this article (10.1007/s40037-020-00635-8) contains supplementary material, which is available to authorized users.

*Patient ownership *is an important element of a physician’s professional responsibility [1]. However, despite considerable research, this concept is incompletely understood, particularly as it relates to responsibilities and expectations for resident physicians in post-graduate training. While numerous articles have articulated the importance of fostering patient ownership among medical trainees [1–5], supervising attending physicians, residents, and medical students can have different expectations of *how* residents should take ownership of their patients’ care and what it *means* to take ownership of a patient’s care [6]. Prior literature has described factors that promote or inhibit patient ownership, including personal attitudes or attributes of the physician, socially-constructed expectations, and environmental factors [6–11]. However, the relative influence of these factors, in addition to specific expectations surrounding patient ownership, is context dependent. Accordingly, understanding specific definitions of and influences surrounding patient ownership in particular practice settings is important for residency programs striving to build clinical experiences that ensure learners understand the concept of patient ownership and display it in practice.

A specific setting in which patient ownership merits further study is continuity clinics, which are outpatient clinics within U.S. primary care residency programs (pediatrics, family medicine, internal medicine) in which residents longitudinally follow a panel of patients as their primary care physician. Several publications have offered behavioral definitions of patient ownership, proposed what it entails (including concepts such as responsibility, knowing patients well, advocacy, autonomy, and communication) [1–6, 12–15] and described how these concepts could be operationalized through a scale of attitudes and behaviors related to ownership of patient care [16]; however, none have been specific to continuity clinics [16]. Furthermore, the unique influencers of patient ownership within continuity clinics have not been fully explored. Several studies have suggested continuity of care facilitates patient ownership [17–21]. However, some characteristics of continuity clinics could poorly position residents to take ownership of their patients’ care, including, for example, competing clinical demands, time constraints, and scheduling limitations [7–11, 22]. Investigating the interplay between these and other factors that might be specific to continuity clinics could inform efforts to optimize clinic operations and structures in ways to promote patient ownership.

Furthermore, other gaps remain in our current theoretical understanding of patient ownership that could be informed by examining this concept within continuity clinics in greater depth. First, because continuity clinics entail sustained engagement with patients over time, they provide an optimal venue in which to incorporate the patient’s voice into our understanding of this concept. Patients have not been included in any research to date specifically examining patient ownership. Much literature has highlighted the active role of the patient and the importance of patient autonomy in relationship building and medical decision-making within the patient–physician relationship [23–26], as well as the importance of continuity of care in fostering this relationship [27, 28]; however, although patient ownership is closely linked to these concepts, some scholars have warned that the very construct of patient ownership entails a hegemonic power differential that is disempowering to patients [29]. We believe it is important to explicitly attend to the patient’s role within the concept of patient ownership and critically examine how this relates to previously described conceptions of patient activation, patient autonomy, and the patient–physician relationship as a whole.

A second theoretical gap is how to reconcile the traditional concept of patient ownership as an individual responsibility with the increasing prominence of team-based care. Most research examining how concepts of patient ownership have changed in light of team-based care has focused on inpatient care and duty-hour restrictions [1, 6, 30–33], with far less attention paid to how such shifts have affected patient ownership in the outpatient setting. The modern focus on team-based care within the outpatient setting must also inform our ongoing investigations into and theoretical understandings of patient ownership.

To address these gaps, we first aimed to provide an in-depth and practical description of patient ownership within continuity clinics. Specifically, our research questions were: (1) What are the definitions, expectations, and experiences of patient ownership from the perspectives of residents, attending physicians, and patients? and (2) What factors promote or inhibit attitudes and behaviors related to patient ownership among residents in continuity clinics? Attending to these questions from a diversity of perspectives should allow for a more representative and robust understanding of patient ownership within continuity clinics. This knowledge can then help clarify expectations for residents and identify specific facilitators or barriers that could be optimized to improve resident education and patient care. Our second aim was to use these findings to build a theory of the concept of patient ownership within continuity clinics.

## Methods

### Setting and participants

This study was conducted within Wright State University/Wright-Patterson Medical Center Pediatric Residency Program. In this integrated program, military residents have a continuity clinic at Wright-Patterson Medical Center, and civilian residents have a continuity clinic at the Dayton Children’s Pediatrics Clinic. Residents and attending physicians were recruited via email from both clinical sites. Given that this research was in a pediatric setting, families of patients seen at each clinic were recruited for participation using informational flyers.

### Data collection and analysis

We employed constructivist grounded theory because this methodology supports the study of complex social phenomena—like patient ownership—that have yet to be theoretically described in detail [34]. While prior literature has provided descriptions and definitions of patient ownership [1–6, 12–15], such work has not been expanded to the level of theory generation and not focused explicitly on this phenomenon within continuity clinics. We approached this topic from a constructivist paradigm [35] because we view patient ownership as a social construct created and agreed upon by an individual patient and physician, and as something which will be influenced by each individual’s prior experiences, personal beliefs, and local context.

From March–August 2019, data collection and analysis took place concurrently across three cycles of semi-structured interviews, with each cycle consisting of interviews with three residents, three attending physicians, and three patient families (one family interview included two parents; Tab. 1), for a total of 27 interviews. The interviewer (MK) asked participants about their definitions, expectations, and experiences of patient ownership and factors that they believed influenced patient ownership (see Electronic Supplementary Material for the interview guides). Interviews were audio recorded and professionally transcribed, with participant identifiers removed.

**Table 1:** Participant characteristics

Participant Group	Total	Military	Female
Residents	9	5 (55%)	7 (78%)
– First-year	3	2 (67%)	2 (67%)
– Second-year	3	1 (33%)	3 (100%)
– Third-year	3	2 (67%)	2 (67%)
Attending physicians	9	5 (55%)	7 (78%)
Patient family members	10	7 (70%)	6 (60%)

Three investigators (MK, HM, LV) engaged in constant comparative analysis, using NVivo Version 12.5.0 software to assist with data coding and analysis. Researchers first independently coded interview transcripts from cycle one, identifying recurring initial codes. The team then met to refine codes and generate preliminary themes. After cycle one, the investigators modified the interview guide to explore the initial themes and probe for gaps. After cycle two, we further refined the theme structure and modified the interview guide to vet and explore the boundaries of the developing themes. We determined that no significant new ideas or themes were emerging after the third round of interviews. After all transcripts were analyzed and final themes were defined and explored, we inductively examined the interconnections between our themes, analyzed memos written throughout the data analysis process, and created visual diagrams to begin constructing a theory describing patient ownership. In refining this theory, we attended to which features of our findings seemed most specific to continuity clinic settings and which we believed to be more overarching principles of patient ownership in general, and we refined our premises and diagram to reflect both the definitions and key influencers identified by our participants.

### Reflexivity

We acknowledge that our educational backgrounds and personal experiences affected our conduct of this study and the meanings we generated. MK is a general pediatrician, attending physician at one of the continuity clinics in our study, and Associate Program Director of the residency program. Her position within the clinic and residency program could have influenced participant responses; however, we emphasized to participants that our questions had no right or wrong answers, and we did not include value-laden questions such as participants’ assessments of how well certain physicians take ownership of patients or families’ impressions of the care they receive at their clinic. Additionally, MK’s professional experience working within a continuity clinic setting could have influenced her analysis of participant responses. LV and HM are educational researchers with expertise in qualitative research and have no direct involvement in the residency program or continuity clinics. All three authors have previously researched this topic as part of a scoping literature review. Although our methods for this study were inductive, we recognize that insights derived from prior research could have informed our understandings.

## Results

We identified four principal themes, each with points of convergence and divergence between the three participant populations. Themes highlighted that participants viewed patient ownership as a relational phenomenon that entails an active role for both the physician and the patient/family, with a supportive role for other members of the medical care team. Continuity of care was generally seen as a facilitator of patient ownership, but other factors mediated this relationship. All participants acknowledged and accepted systems-based barriers to patient ownership within continuity clinics.

### Patient ownership is a relational commitment between patient and physician that includes both affective and task-based components

Participants universally described task-oriented activities that represented patient ownership, including, for example, the physician following up on laboratory or radiology results and making important medical decisions. All participant groups also emphasized that patient ownership involves more than such tasks; it also entails a fundamental desire to connect on a relational level. For the residents and attendings, this connection was expressed as a desire to be seen as the primary physician for patients and to know their patients in a meaningful way:

My definition of patient ownership is that my patients know who their primary care doctor is. In residency, that’s challenging. So, I try to make sure that they know that I am the doctor and they can come to me for anything—even if it’s just questions through the nurse line.—Resident 3

Similarly, patient families communicated a desire to be known and to be heard by their physician.

I’ve appreciated the fact that my provider actually knows my kid, she remembers my kids … I came in for an appointment for my son. … the provider knew my son’s name because he was the patient, but then she started talking to my daughter by her name, too, without me even saying my daughter’s name … she remembered or she took the time to look it up.—Family 2

The relative weight placed on the affective versus task-oriented components of patient ownership varied by participant, but patient families were more likely than physicians to emphasize relational and emotional expectations. For example, physicians often highlighted the benefits to patient care and workflow:

[Knowing patients well] just makes the clinic day better and makes your practice easier. It’s a million times easier to follow up a lab that you ordered and you understand why you did it and what your next steps were. The notes go quicker, the notes can be shorter. It just—I feel like it makes all of clinic better when you have, like, your [patients] that you own.—Resident 6

Alternatively, patient families more commonly expressed how such relationships improved their patient experience:

We’ve had exceptional providers here that are so incredibly patient with [my child] and being able to meet his needs and adjust accordingly based on knowing that he’s going to often react quite negatively to the simplest things. I could definitely see where, you know, with his age and size how somebody could easily interpret his actions as just being a rebelling teenager versus if they didn’t know his past medical history.—Family 5

This difference in emphasis (i.e., physicians focusing on tasks, families focusing on affective experiences) illustrates how each group interpreted the relational commitment of patient ownership differently. Patient families believed that kindness, compassion, and good communication skills were important elements of patient ownership, whereas no attendings or residents described similar perceptions. Attendings and residents instead stressed responsibility for task completion such as care coordination amongst specialists, patient handoffs, and complete documentation in their conceptions of patient ownership. However, all participant groups identified this relational commitment as foundational to the concept of patient ownership, even if its display in action looks different for patients versus physicians.

### Continuity of care exerts a powerful influence on patient ownership, but it cannot guarantee patient ownership

#### Importance of continuity of care

All groups cited continuity of care as the most important facilitator of patient ownership. Continuity of care was articulated as a key driver of the patient–physician relationship, and it was credited with improving patient care, physician satisfaction, and workflow:

I think [continuity of care] helps a lot with patient ownership. … If you see a baby from two weeks all the way up, then you know them so well. … So, I think the more you know your patients, the easier it is to take ownership of their care.—Resident 3

Residents and attendings also noted the educational benefit of following a patient longitudinally to see the progression of the patient’s disease process and the result of treatments. Residents reported seeking out continuity, and attendings encouraged residents to strive for continuity with patients in order to maximize their learning.

[I]t’s one thing to go in, gather a history, think through a differential, say, “Let’s order XYX,” or, “Let’s refer to so-and-so.” And then if you never hear what happens to that patient, you have no idea if your initial working diagnosis was correct. … Learning about how that patient plays out, what that disease process plays out. … I think that’s where a lot of the learning ends up happening.—Attending 6

However, while continuity of care was identified as a facilitator of patient ownership, it was neither necessary nor sufficient to achieve patient ownership. Instead, several factors mediated the relationship between continuity of care and patient ownership.

#### Mediating factors

First, residents recognized that their personal feelings toward patients were key mediators of their sense of patient ownership. Even though residents acknowledged the need to provide good care and follow up to all patients regardless of their personal feelings, having a positive personal connection with families made it more likely they would strive to take ownership of the patient’s care:

There’s a couple of families that I’ve taken ownership on, and part of it is, I think—I just like them so much. They’re such nice people. Not that I don’t take ownership of the patient families that aren’t nice, but the ones that I like, truly felt, “Oh, I want you to come see me and only me” are the ones that you really click on a personal level with.—Resident 1

Conversely, in the case of having difficult interactions with families, continuity of care could make it more challenging for them to feel ownership since each encounter was difficult:

I think if it’s a good relationship, then [continuity of care] is a good factor [supporting patient ownership]. But I think there are times when you’ve seen that patient who’s complex, or the family just has something that you don’t agree with. Then it makes it [taking ownership of the patient] harder and harder.—Resident 4

Second, the complexity of a patient’s medical history and presenting complaint also affected the interplay between continuity of care and patient ownership. Patient families and physicians deemed continuity of care less critical for less medically complex patients and presenting issues:

You know, if it is strep throat, it doesn’t matter. I can see someone. They can do what they need to do and move on. If it is something that you know ahead of time that this is going to be something or that it’s starting to become recurrent … then continuity is really important.—Family 4

Finally, several physicians reported that working in a clinic with a *low* degree of resident continuity of care made them *more* likely to take ownership of clinic patients they saw. They felt a responsibility to ensure patient care was not compromised in a system in which they could not assume a patient’s assigned primary care manager would be meaningfully involved in their care:

You never really have that kind of feeling of consistent continuity, and so I think it gets ingrained that we have to treat everyone kind of like we’ll never see them again, because oftentimes we never will … you can’t just brush it off until the next time you see the kid.—Resident 4

However, personal attributes of the residents also influenced their proactive stance towards patient ownership, including the resident’s own intrinsic sense of responsibility toward patients, their comfort with having the medical knowledge needed to take ownership of a patient’s care in the case of more complex patients, and their career goals (i.e., whether or not they intended to pursue a career in primary care):

Some residents just seem to have a different expectation of themselves within the clinic. And I think that stems more from their own kind of confidence and comfort … There’s kind of the transcendental maturity—above the like, “What am I expected to do?”—Attending 6

### Patient families and physicians harbor idealized conceptions of patient ownership but acknowledge the constraints imposed by logistical and systems-based factors

Residents, attendings, and patient families commonly articulated an ideal of patient ownership in which patients would see their primary care physician for every visit and physicians would personally manage all of their patients’ medical needs. However, all groups accepted that the reality of modern medicine is unlikely to enable the realization of this ideal. Participants identified time constraints, competing responsibilities, and the tension between prioritizing access versus continuity in scheduling practices as barriers to achieving this ideal version of patient ownership:

When I’m just so busy for months in the inpatient side … I would like to be able to take care of [my clinic] patients and feel like they think that I’m their doctor and not just somebody they see every fifth time they come in. … But I do understand that something will have to give during this time frame in order to just complete residency.—Resident 9

While physicians wished they could have more time to connect with patients and be more involved in all aspects of their care, almost all saw these systems-based barriers as an inevitable aspect of residency:

So there is a schedule conflict with the overall residents’ schedules. But again, that is from an over perspective of the entire residency. It’s not their fault, it’s not my fault, it’s just the way it is that’s going right now.—Attending 4

Similarly, patient families displayed understanding of and empathy for physicians working under this system and did not fault them for not living up to the ideal:

I think that it’s nice when you can see the same provider, especially for well [child] visits, because they kind of know the history of the background, but I also understand that they can’t be here 24/7. So, that’s why we have a team that, you know, that reviews the chart, kind of looks through things.—Family 3

In recognition of these constraints, multiple physicians elaborated on systems in place to ensure that patients still received proper care. Patient handoffs, both formal and informal, and relying on other members of the care team, such as nurses, to monitor laboratory and radiology results were key mechanisms to mitigate these barriers. These team-based systems were seen not only as a part of the local context but also as a necessary task related to patient ownership.

A few residents discussed challenges to maintaining patient ownership when personal or systems-based limitations were not acknowledged or accepted. One noted that identifying too much with patients’ concerns or feeling too much responsibility for their care could lead to exhaustion or even burnout:

I think you can go too far [with having patient ownership] in medicine. … I’ve had attendings tell me I need to be careful with empathy versus compassion because you can only empathize so much. … One attending said it really well. He said: “there was a lot of suffering in that room, and you took too much of it.”—Resident 7

Another resident described feeling guilt after coming off of a clinic block and handing off follow-up tasks, even though such handoffs were expected.

When I’m here for a month I try very hard to make sure I’m the only person following on labs that I order. And I always feel really guilty when I have to send them to somebody else because I’m done.—Resident 9

These more extreme expectations of ownership were identified by residents, not attendings. All physicians expected residents to take full ownership of their patients’ care while in clinic, but most did not expect residents to be attending to clinic-related items while on other rotations and expected residents to hand off follow-up tasks more frequently.

### Patient families and physicians hold an expansive view of team-based ownership that includes physicians, support staff, and patient families

Participants described patient ownership as a hybrid of individual- and team-based responsibility. Most saw the physician as personally responsible to patients as the leader of a care team, and no physician thought that the presence of multiple team members diminished this responsibility. Some referred to the physician as the “quarterback” of the team (Attending 9) or even the “commander in chief” (Attending 4). Yet nearly all articulated a team-based concept of patient ownership, with the team including not only other physicians within the clinic, but also nurses, case managers, social workers, front desk staff, and technicians:

First and foremost, [responsibility] goes to the individual [physician], but then the team is there kind of as a backup. So, especially with our teams and our nurses, they do a great job of helping coordinate to get some of that information, get the ED records, get the labs, get everything like that. But then, that decision-making usually comes on the individual.—Attending 2

Additionally, multiple patient families and some physicians identified patients and patient families as integral members of this team. They viewed patient ownership as a shared responsibility between physicians and patient families. Patient families were seen as responsible for advocating for their children, navigating the medical system to ensure proper care and follow up for their child, and negotiating and adhering to medical recommendations made by the physician.

It’s definitely team-based. You can give me a plan of care, but it’s on me, the parent, to carry out that plan. … So, it is you, me on the team together, and my child.—Family 8

As a member of this care team, patient families partnered with the physician in their child’s care. For patients and physicians who espoused this joint concept of patient ownership, they described the patient–physician relationship as a partnership, each with distinct roles and responsibilities.

Patients that I have kind of taken ownership of work to be in my clinic. They seek me out, they book all their well visits with me, they will try to book acute visits with me, they’ll adjust their schedules to try to be with me.—Resident 6

Patient ownership I think definitely revolves around for me the word relationship. I think patient ownership, I think about health. I think that ownership has to be 50–50. Ownership of health has to be patient and physician.—Resident 7

This central position for the patient/family within the medical care team aligned with the bi-directional relationship articulated by participants in defining patient ownership. Both sets of descriptions cast active and empowered roles for patients/families within the construct of patient ownership, which became a foundational premise of our theory of patient ownership within continuity clinics.

## Discussion

**Fig. 1:**
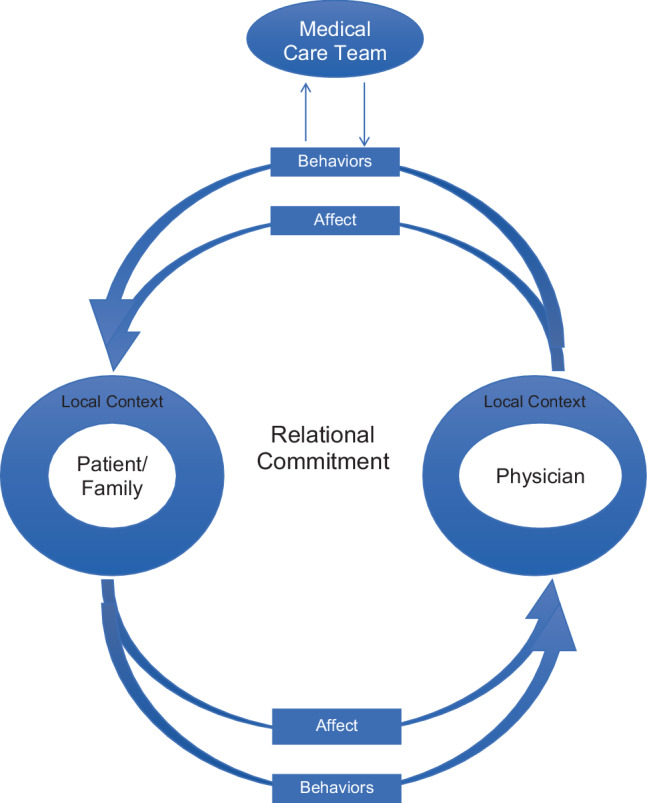
Visual representation of theory of patient ownership within continuity clinics

Our findings provide new insights into the concept of patient ownership within continuity clinics by incorporating the perspectives of attending physicians, residents, and patient families. We developed a theory of patient ownership within continuity clinics that rests on several premises, illustrated in Fig. 1. The first premise of our theory is: *Patient ownership within continuity clinics is a relational commitment that involves a bi-directional exchange between patient and physician*. This relationship exists within the context of medical care provision, which is the foundational task upon which this relationship is built. For both parties, patient ownership entails distinct affective and behavioral elements. Tab. 2 provides examples of such elements within our continuity clinic setting. The attitudes and behaviors articulated for physicians align well with prior descriptions of patient ownership that emphasize feelings of responsibility, personally carrying out patient care tasks, autonomy, and knowing patients well [5, 8, 12, 13, 15, 29, 36, 37]. Within our continuity clinics, coordination of care within a team-based environment was emphasized as a particularly important task, including involvement of medical support staff and patient handoffs to other physicians.

**Table 2:** Examples of affective and behavioral elements of patient ownership in a continuity clinic setting

	Affect	Behaviors
Physician	**–** Feels responsibility for patient’s care **–** Feels like primary care provider for patient **–** Sees the patient’s concerns as their own	**–** Provides medical care **–** Follows up clinical care, radiology, laboratory studies, and consults **–** Coordinates care between medical care team and subspecialists **–** Listens to patient **–** Knows the patient well, both medically and personally **–** Makes important care decisions **–** Seeks continuity with patients
Patient/Patient family	**–** Identifies physician as primary care provider **–** Feels known by physician **–** Feels heard by physician **–** Trusts physician	**–** Advocates for self/patient **–** Carries out home treatment plans **–** Actively seeks out appointments/continuity with physician

The second premise of our theory is: *The physician and patient engage in patient ownership within the constraints and affordances of their local context*. We view the systems-based and logistical constraints articulated by our participants (Box 1) as examples of barriers within our local context. Conversely, continuity of care, scheduling practices favoring continuity over access, and resident autonomy were facilitators in most circumstances (Box 1). While many of these factors also align with prior literature describing influencers of patient ownership [7–11, 17–22], findings specific to the continuity clinic setting that have not been well described previously included protocols for calling back patients regarding laboratory or radiology results, the resident patient load during clinic, and the medical complexity of resident patient empanelments. The practice setting and medical care team can also be seen as integral parts of the local context; however, none of these contextual factors can guarantee or disallow patient ownership. The ownership exhibited and felt by both physicians and patients is influenced by, but not dependent on, the context in which they are operating.

### Box 1 Contextual factors affecting patient ownership in continuity clinic setting

- Degree of continuity of care
- Clinic scheduling practices (e.g., prioritizing access versus continuity)
- Resident rotation scheduling practices (e.g., full-day versus half-day continuity clinics, division of inpatient/outpatient rotations)
- Composition of medical care team
- Protocols for calling patients back regarding laboratory or radiology results
- Degree of resident autonomy
- Faculty role modeling
- Resident patient load during clinic
- Medical complexity of patient empanelment
- Handoff protocols/practices

The final premise of our theory is: *A patient ownership relationship is built between physician and patient. Therefore, although a physician is part of a care team, this team membership does not diminish a physician’s personal commitment toward his/her patients*. Prior literature has expressed concern over a dilution of responsibility among residents in light of team-based care [2–4], but no participants within our study felt that team-based care lessened personal feelings of patient ownership. Instead, care teams played an important role in facilitating provision of care, attending to follow-up issues, and coordinating additional care.

We contend that an important contribution offered by our theory is to articulate the active role cast for the patient/family within the construct of patient ownership. In our theory, the patient is not the passive recipient of a physician’s care. Instead, the patient has agency to actively partner with the physician in negotiating and carrying out medical care plans, and the feelings the patient has toward his/her physician matter. Of course, the physician can still execute his/her responsibilities within this relationship even if, for example, the patient/family does not adhere to treatment plans. But can a physician really be seen to have good ownership of a patient’s care if the patient/family does not feel heard or listened to, or if they do not see that particular physician as *their* physician?

Within the larger body of literature on the patient–physician relationship, patient autonomy and activation are increasingly recognized as central concerns [23, 26, 38, 39]. Emanuel and Emanuel [40] described four prototypical models of this relationship: paternalistic (strong control exerted by physician), informative (physician only provides information, patients have full control of decisions), interpretive (physician helps patient elucidate and articulate their values), and deliberative (physician partners with patient to decide treatment plan based on patient values), with the conception of patient autonomy as one of the prime distinguishing features of each. Some have contended that the term *patient ownership* is inherently paternalistic [29] and suggested alternative phrasings such *patient care ownership* or *decision ownership* [2, 8, 36], and we respect those who chose to embrace different terminology. However, this model reframes patient ownership in a more inclusive sense by shifting the locus of control from the physician alone to a shared position between both parties—with the physician still having information and expertise, and with the patient/family empowered to negotiate and carry out treatment plans and to shape the nature of the relationship through their feelings toward the physician. We suggest that our theory of patient ownership is compatible with a more interpretive or deliberative model of the patient–physician relationship, which some have argued to be the most ethical models of interaction [38] and the most likely to foster trust [39, 41], and it brings this concept in line with more modern views of patient autonomy.

We acknowledge our study has limitations. In accordance with constructivist epistemology, our findings are specific to our particular context and shaped by our subjective perspectives. Further study is needed to determine the transferability of our theory to clinical settings other than continuity clinics. We suspect that the longitudinal, sustained relationships built within continuity clinics—the interpersonal facet of continuity of care [42]—could exert a strong influence on expectations surrounding the patient/family role within the patient ownership relationship. How the patient/family role is articulated and experienced in inpatient or other acute care settings, for example, merits investigation. Furthermore, the patient families who agreed to participate in the study were predominantly military (70%). While we did not notice significant differences in responses between groups, we recognize that factors such as frequent moves within this population could affect participant expectations regarding patient ownership and continuity of care. We also acknowledge the strong female predominance among resident and attending physician participants, which mirrors the gender distribution of the program’s residents and clinic attending physicians.

We hope future studies can build upon these findings by vetting this theory in other settings. Using contextual factors identified in this study, future work could also examine how to reshape clinical policies or practices designed to optimize patient ownership between residents and patients in continuity clinics, particularly in light of the unique time and systems-based barriers they face in training. In all future studies of patient ownership, we hope this theory can reframe the concept in a more inclusive light that continues moving these important conversations forward in a way that respects the diversity of perspectives involved.

## Caption Electronic Supplementary Material

Interview guides for attending physician, resident, and patient family interviews
