# Supplementary material for: “It is you, me on the team together, and my child”: Attending, resident, and patient family perspectives on patient ownership
Source: Perspect Med Educ. 2020 Dec 2;10(2):101–9. doi: 10.1007/s40037-020-00635-8 (PMC7952476; doi:10.1007/s40037-020-00635-8)
Supplement: Supplementary file 1 — Interview guides for attending physician, resident, and patient family interviews [file 40037_2020_635_MOESM1_ESM.docx]

This Electronic Supplementary Material consists of three patient ownership interview guides: for residents, attending physicians, and patient families.

Patient Ownership *Resident* Interview Guide

Consent Script: Thank you for participating in this interview. Before we begin, I’d like to confirm that you are in agreement with moving forward with the interview. Please reply yes or no to the following two questions:

1. Do you agree to participate in this study of patient ownership as it was described in the information you received from the research team via email?
2. Do you agree to have this interview audio recorded, knowing that all identifying information will be removed during the transcription process and therefore removed before any member of the research team reviews the data?

As a reminder, we will be audio recording the interview, and I will begin the recording now.

Introduction Script: The goal of this interview is to hear your perspectives on issues related to the concept of patient ownership. Since there is not one generally accepted definition of this term, part of what we are hoping to do with these interviews is to find out what it means to different people. As we are going through these questions, we want to respect the privacy of any patients, families, and staff members involved. Therefore, we ask you not to use the names of particular patients, doctors, or other staff members. We ask you to be as honest and open in your responses as possible, but do not feel pressured to answer any question or share any information that makes you uncomfortable. The types of questions we will be discussing have no right or wrong answers. Do you have any other questions before we start?

Before we begin the interview questions, I have a few demographic questions to begin.

1. Can you please state your participant number?
2. Can you please state your year of training?

Thank you. We will now begin with the interview questions.

1. What does patient ownership mean to you?

*Rephrasing if needed*: How would you define patient ownership?

1. What factor or factors do you think have been most important in shaping your concept of patient ownership?

*Probes*: Do you remember when you first heard this term? If so, in what context? Are there particular people in your past who have been influential in modeling patient ownership for you? Were there particular expectations surrounding patient ownership in your training that influenced your views?

1. Can you tell me about a physician who displayed good patient ownership? As a reminder, please do not use anyone’s name in your response.

*Probe*s: What did they do? What specific behaviors did you see? In general, what specific behaviors taken by a physician would indicate they are displaying good patient ownership?

1. What expectations do you think are reasonable have of a resident in terms of following up issues that arise during a visit? Does this differ from your expectations for an attending?

*Probes*: How important is it to you to personally follow up laboratory results? …radiology studies? …results of consults placed? Is it OK with you if this task is handed off to another provider as long as it is done? Do you think there should be different expectations if you are seeing a patient who is empaneled to you versus one who is empaneled to another provider? Do you think that these expectations stem from yourself, your program, or your attendings?

1. To what degree do you think the expectations outlined above stem from yourself, your program, your colleagues, and/or other factors?

*Rephrasing if needed*: Do you think your colleagues share your expectations? Do you think different attendings within our program have similar expectations of you? What expectations do you think our program, as a whole, makes explicit to the residents?

1. To what degree do you think your attitudes regarding patient ownership stem from your own intrinsic sense of responsibility, residency programmatic expectations, systemic factors (such as scheduling concerns), and/or other factors?
2. How frequently do you think you are able to fulfill the expectations we just discussed regarding patient ownership?
3. What factors do you think facilitate you being able to practice good patient ownership?

*Rephrasing if needed*: What factors make it more likely that you are able to fulfill the expectations we discussed above?

1. What factors do you see as barriers to practicing good patient ownership?

*Rephrasing if needed*: What factors make it less likely that you are able to fulfill these expectations we discussed above?

1. To what degree do you think continuity of care influences your attitudes and behaviors related to patient ownership?
2. To what degree do you see patient ownership as a team-based responsibility versus the responsibility of an individual provider?

*If more of a personal responsibility*: Why? What role do you see nurses or technicians play in ensuring good patient ownership?

*If more of a team-based responsibility*: Why? Do you think this leads to a diffusion of responsibility among residents?

1. Do you have any personal experience in which you felt you (or another resident) did an excellent job taking ownership of a patient? Please do not use names in your response.

*Probes*: If so, what stood out to you about this interaction? Do you think it is reasonable for a provider to always try to display this degree of patient ownership, or was this an exception? How did the experience shape your view of your relationship with that patient? Do you see any systemic factors (such as scheduling access, communication methods, or team structures) that could have contributed to this experience?

1. Do you have any personal experiences in which you felt you (or another resident) did a poor job taking ownership of a patient? Again, please do not use any names.

*Probes*: If so, what stood out to you about this interaction? Was this an anomaly, or is this a problem you have encountered more frequently? Do you see any systemic factors that might have influenced this experience?

Summary Questions:

In this last question set, I would like to ask you a few summary questions.

1. Before we end the interview, I want to make sure you had a chance to share all of your thoughts and opinions. Is there something we didn’t talk about that you think is important?
2. Is there anything else you would like to add?
3. Would you be willing to be contacted by our research team for further clarification on your answers?

On behalf of the research team, I’d like to thank you for participating in this interview.

Patient Ownership *Attending* Interview Guide

Consent Script: Thank you for participating in this interview. Before we begin, I’d like to confirm that you are in agreement with moving forward with the interview. Please reply yes or no to the following two questions:

1. Do you agree to participate in this study of patient ownership as it was described in the information you received from the research team via email?
2. Do you agree to have this interview audio recorded, knowing that all identifying information will be removed during the transcription process and therefore removed before any member of the research team reviews the data?

As a reminder, we are going to be audio recording the interview, and I will begin the recording now.

Introduction Script: The goal of this interview is to hear your perspectives on issues related to the concept of patient ownership. Since there is not one generally accepted definition of this term, part of what we are hoping to do with these interviews is to find out what it means to different people. As we are going through these questions, we want to respect the privacy of any patients, families, and staff members involved. Therefore, we ask you not to use the names of particular patients, doctors, or other staff members. We ask you to be as honest and open in your responses as possible, but do not feel pressured to answer any question or share any information that makes you uncomfortable. The types of questions we will be discussing have no right or wrong answers. Do you have any other questions before we start?

Before we begin the interview questions, I have a few demographic questions to begin.

1. Can you please state your participant number?
2. Can you please share your role as it relates to teaching pediatric residents in a continuity clinic setting?

Thank you. We will now begin with the interview questions.

1. What does patient ownership mean to you?

*Rephrasing if needed*: How would you define patient ownership?

1. What factor or factors do you think have been most important in shaping your concept of patient ownership?

*Probes*: Do you remember when you first heard this term? If so, in what context? Are there particular people in your past who have been influential in modeling patient ownership for you? Were there particular expectations surrounding patient ownership in your training that influenced your views?

1. Can you tell me about a physician who displayed good patient ownership? As a reminder, please do not use any names in your responses.

*Probe*s: What did they do? What specific behaviors did you see? In general, what specific behaviors taken by a physician would indicate they are displaying good patient ownership?

1. What expectations do you think are reasonable to have of a resident in terms of following up issues that arise during a visit? Do these expectations differ from your expectations for an attending?

*Probes*: How important is it to you that a resident personally follows up laboratory results? …radiology studies? …results of consults placed? Is it OK with you if this task is handed off to another provider as long as it is done? Do you think there should be different expectations if residents are seeing a patient who is empaneled to them you versus one who is empaneled to another provider?

1. To what degree do you think the expectations outlined above stem from yourself, your program, your colleagues, and/or other factors?

*Rephrasing if needed*: Do you think your colleagues share your expectations? What expectations do you think our program, as a whole, makes explicit to the residents?

1. To what degree do you think the attitudes residents have regarding patient ownership stem from their own intrinsic sense of responsibility, residency programmatic expectations, systemic factors (such as scheduling concerns), and/or other factors?
2. How frequently do you think residents are able to fulfill the expectations we just discussed regarding patient ownership?
3. What factors do you think facilitate residents being able to practice good patient ownership?

*Rephrasing if needed*: What factors make it more likely that they are able to fulfill these expectations we discussed above?

1. What factors do you see as barriers to residents practicing good patient ownership?

*Rephrasing if needed*: What factors make it less likely resident can fulfill these expectations we discussed above?

1. To what degree do you think continuity of care influences resident attitudes and behaviors related to patient ownership?
2. To what degree do you see patient ownership as a team-based responsibility versus the responsibility of an individual provider?

*If more of a personal responsibility*: Why? What role do you see nurses or technicians play in ensuring good patient ownership?

*If more of a team-based responsibility*: Why? Do you think this leads to a diffusion of responsibility among residents?

1. Do you have any personal experiences in which you felt a resident did an excellent job taking ownership of a patient? Please do not use any names in your response.

*Probes*: If so, what stood out to you about this interaction? Do you think it is reasonable for a resident to always try to display this degree of patient ownership, or was this an exception? Do you see any systemic factors (such as scheduling access, communication methods, or team structures) that could have contributed to this experience?

1. Do you have any personal experiences in which you felt a resident did a poor job taking ownership of a patient? Again, please do not use any names.

*Probes*: If so, what stood out to you about this interaction? Was this an anomaly, or is this a problem you have encountered more frequently? Do you see any systemic factors that might have influenced this experience?

Summary Questions:

In this last question set, I would like to ask you a few summary questions.

1. Before we end the interview, I want to make sure you had a chance to share all of your thoughts and opinions. Is there something we didn’t talk about that you think is important?
2. Is there anything else you would like to add?
3. Would you be willing to be contacted by our research team for further clarification on your answers?

On behalf of the research team, I’d like to thank you for participating in this interview.

Patient Ownership *Patient Family* Interview Guide

Consent Script: Thank you for participating in this interview. Before we begin, I’d like to confirm that you are in agreement with moving forward with the interview. Please remember that you can chose to not participate at any time, and this decision will not affect your future relations with Wright-Patterson Medical Center or Dayton Children’s. Please reply yes or no to the following two questions:

1. Do you agree to participate in this study of patient ownership as it was described in the information you received from the research team via email?
2. Do you agree to have this interview audio recorded, knowing that all identifying information will be removed during the transcription process and therefore removed before any member of the research team reviews the data?

As a reminder, we are going to be audio recording this interview, and I will begin recording now.

Introduction Script: The goal of this interview is to hear your perspectives on issues related to the concept of patient ownership. You may be more or less familiar with this term. Since there is not one generally accepted definition of this term, part of what we are hoping to do with these interviews is to find out what it means to different people. As we are going through these questions, we want to respect the privacy of any patients, families, and staff members involved. Therefore, we ask you not to use the names of particular patients, doctors, or other staff members. We ask you to be as honest and open in your responses as possible, but do not feel pressured to answer any question or share any information that makes you uncomfortable. The types of questions we will be discussing have no right or wrong answers. Do you have any other questions before we start?

Before we begin the interview questions, I have a few demographic questions to begin.

1. Can you please state your participant number?
2. Can you please share whether you receive your care at the Wright-Patterson Pediatric Clinic or the Dayton Children’s Hospital Child Health Clinic?
3. Can you please state whether your primary care provider/manager is a resident or an attending physician?

Thank you. We will now begin with the interview questions.

1. As I mentioned earlier, there isn’t a clear definition of patient ownership in the literature. So, I’d like to start by asking you: What does patient ownership mean to you?

*Rephrasing if needed*: How would you define patient ownership?

*Probes*: Have any of you heard this term before? If so, in what context and what did you understand that term to mean or refer to?

1. What specific behaviors taken by a physician would indicate he or she is displaying *good* patient ownership?
2. What expectations do you have of a physician you see at a particular clinic visit in terms of following up issues that arise *during that visit*?

*Probes*: Is it important to you that your provider personally follows up laboratory results? …radiology studies? …results of consults placed? Or it is OK with you if this task is handed off to another provider as long as it is done?

1. Do you have different expectations for follow up if you see your Primary Care Manager (PCM) versus another provider for a particular visit? Do they differ if you see a resident versus an attending?

*Rephrasing if needed*: Do you expect a different degree of follow up for issues that arise if you see your PCM versus another provider? …a resident versus an attending?

*Probe*: Have you seen any actual differences in the degree of follow up you receive when seeing your PCM versus another provider? …a resident versus an attending?

1. Can you tell me about a time in which you felt your provider displayed excellent patient ownership? As a reminder, please do not use any names in your response.

*Probes*: If so, what stood out to you about their actions? Was it a resident or an attending? Do you feel that they went above and beyond in this instance, or is this what you expect of your provider? How did the experience shape your view of that particular provider? How did the experience shape your view of your relationship with your provider?

1. Can you tell me about a time when you felt your provider displayed poor patient ownership? Again, please do not use any names.

*Probes*: If so, what stood out to you about their actions? Was it a resident or an attending? Was this an anomaly, or is this a problem you have encountered more frequently? How did the experience shape your view of that particular provider? How did the experience shape your view of your relationship with your provider? Can you tell me about any other experiences with that provider that you felt were influenced by this interaction?

1. Thanks for sharing your experience. Thinking back over the stories you shared, do you see any system-level or organization-level factors that could have contributed to this experience? So, such factors might be the scheduling access, communication methods, or team structures – but these are just examples. What do you think, at a system- or organization-level, are factors that contribute to patient ownership?

*If yes*: What factors specifically? How do you think they contributed?

*If no:* Why not?

1. Continuity of care is essentially having one consistent doctor manage your health care, or seeing the same doctor for all of your visits. How important is it to you that you have continuity of care in terms of seeing the same doctor at each visit?

*If important*: Why? What benefits do you see from having continuity of care?

*If not*: Why not? Are there other factors you value more in your care?

Have you found that seeing a resident versus an attending influences the degree of continuity of care you have experienced?

1. To what degree do you see patient ownership as a team-based responsibility versus the responsibility of an individual provider?

*Probes*: What role do you see nurses or technicians play in ensuring good patient ownership? If you see this issue as a team-based responsibility, does that change the way you view your relationship with your provider?

Summary Questions:

In this last question set, I would like to ask you a few summary questions.

1. Before we end the focus group, I want to make sure you had a chance to share all of your thoughts and opinions. Is there something we didn’t talk about that you think is important?
2. Is there anything else you would like to add?
3. Would you be willing to be contacted by our research team for further clarification on your answers?

On behalf of the research team, I’d like to thank you for participating in this focus group.
